# Supplementary material for: Oral Administration of Bacillus subtilis Subunit Vaccine Significantly Enhances the Immune Protection of Grass Carp against GCRV-II Infection
Source: Viruses. 2021 Dec 24;14(1):30. doi: 10.3390/v14010030 (PMC8779733; doi:10.3390/v14010030)
Supplement: Supplementary file 1 [file viruses-14-00030-s001.zip › viruses-1457531-supplementary.pdf]

viruses MDPI

Supplementary Materials:

Table S1. Primer sequences in this study.

Gene	Primer	Oligonucleotide Sequence (5′-3′)	GenBank
VP56-1	Forward	CGGGATCCATGAGCCGCATGACTACAGAGCA	GU350744.1
	Reverse	CCCTCGAGTCAATGGTGATGGTGATGATGAT-TCATACTGGCGGTGTTGG	
VP56-2-	Forward	CGGGATCCATGAATGCGACGAGAATAGATGC	
	Reverse	CCCTCGAG-TCAATGGTGATGGTGATGATGTCTAAC-GCTCACCGTCTTTT	
VP56-3-	Forward	CGGGATCCATGTT-GAACAAGTCCCAAGGAAAAT	
	Reverse	CCCTCGAG-TCAATGGTGATGGTGATGATGGACAGTT-GTAATCGTCGGTTCC	
VP56-4	Forward	CGGGATCCATGGCGATAACCACCACTACGGC	
	Reverse	CCCTCGAGTCAATGGTGATGGTGATGATGAT-TACGCAGACCAGCCCACT	
VP56-Q	Forward	CGGGATCCATGGCCACCGTGACAGCC	
	Reverse	GGCTCGAG-TTAATGGTGATGGTGATGATGCTTACAG-CAAATTACCGTCC	
CotC	Forward	CAGGTCGACTCTAGAGGATCCCATGTGTAG-GATAAATCGTTTGGGC	
	Reverse	ATAAGAGCAGCGTAAGTAGGATCCTTAGTAG-TGTTTTTTATGCTTTTTATAC	
VP56-2	Forward *	CGGGATCCATGAATGCGACGAGAATAGATGC	
	Reverse*	CCGGTAC-CTCAATGGTGATGGTGATGATGTCTAAC-GCTCACCGTCTTTT	
IL-1β	Forward	ACTGGAGATGTCTTCGCATCC	AB757757.1
	Reverse	GCAGCGCATCTTCACAAATCT	
MHC-II	Forward	TACTACCAGATTCACTCGG	JF436931.1
	Reverse	CGGGTTCCAGTCAAAGAT	
IFN1	Forward	GACTGCGCAACACATGATGG	JX 657682.1
	Reverse	CTCGTCGATGCTCTTGTCCA	
TNF-α	Forward	AAGTCATAGGTCGAGGTCAGGG	AY303809.1
	Reverse	CGTTTTCACCTTCAATTAGCAGA	
IgM	Forward	GTCAATCTTCGGCTTGTCTCA	DQ417927.1
	Reverse	GGGTATAATCTCCATCGGGTC	
18S rRNA	Forward	ATTTCCGACACGGAGAGG	EU047719
	Reverse	CATGGGTTTAGGATACGCTC	

* The primers were used to construct recombinant plasmid of *B. subtilis.*

Table S2. Feed formulation.

Material Name	Concentration (%)	Material Name	Concentration (%)
Fish meal	18	Blood meal	3

Cottonseed meal	18	The shrimp shell powder	3
Rapeseed dregs	16	Soybean oil + Vegetable Oil (1:1)	2
Powder	10.8	Zeolite powder	2
Soybean meal	10	Calcium dihydrogen phosphate	2.2
Corn	9	Premix	1
Rice bran	5		

Table S3. Statistical results of histological lesions in spleen and trunk kidney of grass carp on day 7 post-GCRV II infection.

	**Spleen**		**Trunk Kidney**	
Lesion	Hemosiderin agglutination	Hypertrophied nuclei	Vacuolization	Hemosiderin agglutination
Health grass crap	-	-	-	-
B. s-CotC-VP56-2	+	-	-	+
B. s-CotC	+ + +	+ + +	+ + +	+ + +
Bacillus subtilis	+ +	+ + +	-	+ +
Control	+ + +	+ + +	+ + +	+ + +

−, none; +, mild; ++, moderate; +++, severe. n = 3.

Table S4. Mortality rate and RPS of grass carp challenged.

**Group**	**Death/Days**								**Mortality Rate (%)**	**Survival Rate (%)**	**Relative Percent Survival (RPS) (%)**
	**1**	**2**	**3**	**4**	**5**	**6**	**7**	**8**			
Control/	0	0	4	5	7	8	15	11	100	0	-
Bacillus subtilis	0	0	3	1	4	12	13	7	82	18	18
B. s-CotC	0	0	1	3	8	11	10	6	79	21	21
B. s-CotC-VP56-2	0	0	1	1	3	4	7	5	44	56	56

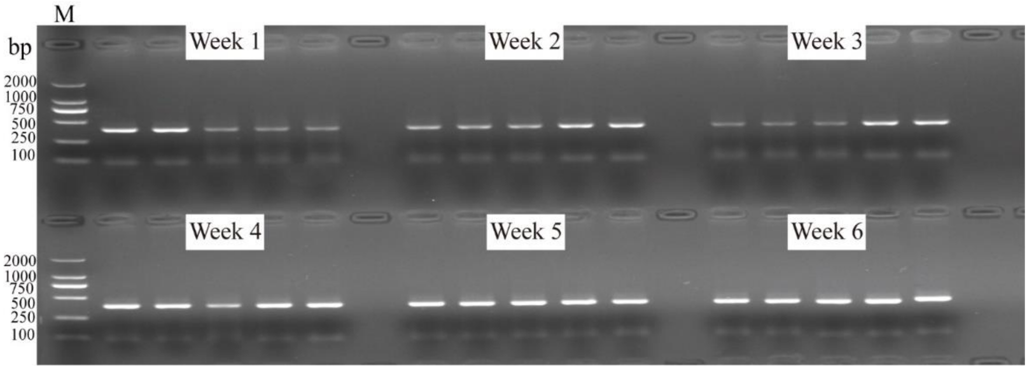

Figure S1. PCR detection of spore colonization in the intestine of grass carp. Lane M: DNA Marker; Detect 5 fish every week, each well is the PCR result of a mixture of 10 colonies.
